# Community Health Impact Assessment in Ghana: Contemporary Concepts and Practical Methods

**DOI:** 10.1177/0046958019845292

**Published:** 2019-06-17

**Authors:** Da-Costa Aboagye, Kwame Akuffo, Hafiz T. A. Khan

**Affiliations:** 1 University of West London, UK

**Keywords:** community health impact assessment, contemporary concepts, practical methods, National Health Insurance Scheme, research, public health

## Abstract

It has long been recognized that health and its determinants are strongly influenced by policies, programs, and projects outside of the health care sector. Few countries have introduced health impact assessments (HIA) to try and ensure that probable impacts on health are considered. An appropriate health impact assessment regime will identify negative and positive impacts of proposed health policies and programs on health, enable the interpretation of health risk and potential health gain, and present the information to assist in decision making. These HIAs are often generic and rapid desk–based appraisals characterized by the use of information and evidence that is already available or easily accessible and generally undertaken by administrators in an organization to gain a snapshot of the health impacts to inform proposal direction. Rapid and generic desk–based assessments require less-intensive effort and resources and draws on existing data sources from scientific peer-reviewed and gray literature to analyze potential health impacts. However, both sources can also be used to determining whether a more detailed review is necessary. The Community HIA model proposed by this work departs from the generic and rapid desk–based appraisals and is intended to provide practical evidence to give higher priority to people’s viewpoints, promote participation, understanding and incorporate community voices to help shape future policy, programs, and practice. A comprehensive review of Ghana’s National Health Insurance Scheme (NHIS) was carried out using the generic desk–based HIA approach. This was followed by a practical qualitative community field work. In this research, we have demonstrated how community HIA is to be conducted through an actual case study in the Ghanaian West African context. The scope of this work is wide and incorporates the consideration of key concepts and possible methods for carrying out HIA at the community level.

## Introduction

Health impact assessment (HIA) is a fast-emerging contemporary process. This article aims to contribute to an understanding of community HIA in Ghana and also to demonstrate how impact assessment might be practically conducted in such an environment of limited health development and scarce resources. The scope of this work covers some of the key concepts related to impact assessment and possible methods that may be more appropriately and effectively applied at the community level. It also analyzes how community participation may be promoted based on understanding the voices of ordinary people in making informed policy decisions. In short, how best to recognize community perspectives in policy planning and implementation to achieve maximum impact at the affordable cost.

This work is aimed primarily at policymakers and attempts to provide an approach that departs from the standard generic desk–based HIA because of its limitations, in favor of a more practical and pragmatic approach of community HIA, better suited to African, or indeed, developing countries generally.

## Community HIA—Provenance and Purpose

The public health challenges of the 21st century are extremely complex. Thus, the solutions require sophisticated and comprehensive approaches that transcend the narrow bounds of the health sector itself, bringing together partners across other policy sectors and also community action.^1^ Howard and Gunther^2^ noted that community actions lie outside the mainstream health sectors and government policy development objectives are best achieved when all sectors including community actions are collaborative. This is because, by definition, health issues intersect all aspects of social existence. The effective use of healthy public policy approaches therefore relies strongly on HIA.^3^

The HIA process advocated by this article is embracive and therefore, when assessing a proposal, takes on board relevant evidence that includes the views of the public/community which is considered alongside expert opinion and scientific data, with each source of information being valued equally within the HIA.^4^

This embracive approach is argued to be so critical to HIA that it is advanced as the foundation upon which the HIA approach advocated by this work has been based. HIAs are good tools to assess health policies in terms of impact on health. Communities must therefore be part of HIAs and this fact is now internationally accepted as the framework used in policy evaluations.^5^ In this context, it is important to point out that an HIA does not make decisions; it is a methodological approach by which the best information is presented to decision makers in a clear and transparent way.^6^ Unfortunately, as indicated above, most HIAs have been solely desk-based with no lay community perspectives. Even more serious is the lack of evidence of the use of any kind of HIA in policy assessments in Sub-Saharan Africa, despite the undoubted flexibility, simplicity, transparency and cost-effectiveness, acceptance and explicit success in carrying out local, regional, national, and international policy analyses in developed countries.^7^ The aim of this article, therefore, is to contribute to meeting this gap and focuses on HIA concepts and practical methods using an example from a study conducted in Ghana, West Africa. The study used HIA framework to examine a National Health Insurance Scheme (NHIS) in Ghana. This article describes the methods used and discusses how HIA is a useful tool for ensuring progressive understanding and avoiding retrogression in pro-poor policies such as the national health insurance financing policy in Ghana.

## Conceptual Framework

Most policy procedures are prepared in the framework of methods or approaches that may be more or less specific in a given population and at a given period. It is vital that such approaches or methods are taken into account, otherwise community HIA runs the peril of being a non-natural process, separated from the realism of the policy contexts in which it is being executed. This proposed method suggests that in addition to promoting the utmost health of communities, 5 determinants are predominantly essential for community HIA as detailed in Table 1.

**Table 1.** Authors’ Construct.

Sustainability	Emphasizing long- and short-term impacts as key to decision making.^8^
Human Rights	Emphasizing the right of people to participate in a transparent process for the formulation, implementation, and evaluation of policies that affect their lives, both directly and through the elected political decision makers.^9^
Equity	Emphasizing that community HIA is not only interested in the aggregate impact of the assessed policy on the health of a population but also on the distribution of the impact within the population, in terms of gender, age, ethnic background, and socioeconomic status.^10^
Ethical application of evidence	Emphasizing that the use of Quantitative and Qualitative, community participatory evidence has to be rigorous and based on different scientific disciplines and methodologies to get as comprehensive assessment as possible of the expected impacts.
Collaboration	Emphasizing multisectoral approaches. Intersector actions within and outside public and private sectors and community views are emphasized.^11^

## Sustainability

The concept of health promoted by advocates of HIA is a broad one. Sustainability is regarded as necessary conditions for health and development, as exemplified by the Sustainable Development Goals. Sustainability may be defined as meeting the needs of demand of communities without compromising the ability of future communities to meet their own needs.^8^

## HIA, Human Rights, and Equity

It is contended that any contemporary discourse on health policy promotion must necessarily engage human rights. The Constitution of the World Health Organization (WHO) envisages “ . . . the highest attainable standard of health as a fundamental right of every human being.”^12^ WHO provides the following cogent justification:

Understanding health as a human right creates a legal obligation on states to ensure access to timely, acceptable, and affordable health care of appropriate quality as well as to providing for the underlying determinants of health, such as safe and potable water, sanitation, food, housing, health-related information and education, and gender equality.^13^

The foundation instruments of the United Nations system for the international protection of rights are the Universal Declaration of Human Rights (UDHR),^9^ the International Covenant on Civil and Political Rights (ICCPR),^14^ and the International Covenant on Economic. International Covenant on Economic Social and Cultural Rights (ICESCR)^15^ must be read together as an interlocking normative system in which rights are “ . . . universal, indivisible and interdependent and interrelated” (para 5, Vienna Declaration on Human Rights and Programme of Action).^16^ Since human rights are not meaningful to a person who is afflicted by poor health, if occasioned by want of the material necessities of life as affirmed by the ICESCR. This is the reason why Krieger et al^17^ highlight that HIA and human rights are both associated with the promotion of health and well-being. In addition, the principle that human rights are universal and inherent in all human beings as propagated by the UDHR and the Vienna Declaration and the WHO principles indicated above are the reasons why effective HIA is essential in ensuring the institution of good health policies and programs to facilitate at least, the minimum well-being of all without reference to their socioeconomic status in a world in which the health outcomes for the rich and poor are poles apart—according to United Nations research statistics, the average life expectancy in the highest developed economies is 80 years and 59 years in the lowest developed countries.^18^

Health impact assessment is an essential tool for understanding health inequality as it is able to assess the community impact of proposed policies, plans, or projects on communities.^19^ Thus, for HIA, equality or equity in well-being is a core value and many specialists have employed HIA to advance equity in decision-making processes.^20,21^ However, as the field becomes better known, there is a danger that the concentration on equity will diminish without explicit attention, care, and guidance regarding its role in HIA practice.^22^ Inequity has a moral and ethical dimension, resulting from avoidable and unjust differentials in health status.^23^ Equity in health entails that ideally everybody should have a fair opportunity to attain their full health potential and, more plausibly, that no one should be disadvantaged from achieving this potential if it can be avoided.^10^

## Collaboration

Intersectoral actions within and outside public and private sectors and community views are emphasized. For example, the health sector cannot achieve health-related goals as an isolated, stand-alone system.^11^ It needs the involvement of local leaders who govern by proximity and ensure the implementation of social policies, the transportation sector builds reliable roads that can decrease transit time for ambulances and cars to reach hospitals, the communications sector helps promote the existence of new health care services, and so forth.^11^ This means that improving inter-sector cooperation can support programs that promote working together across various sectors.

## Ethical Application of Evidence

Depending on the type of HIA being undertaken, either community participatory approaches, qualitative or quantitative methods or both can fit within HIA framework. This article is focused on how lay community views fit within the HIA framework. In other words, it discusses how focus group discussions (FGDs) and key informant interviews can be an integral part of HIA framework to bring the views of people at the community level.^24^ The FGDs and key informant interviews present the actual perspectives.^25,26^ The community HIA being proposed used key informants and focus group participants and are discussed as part of how to carry out a community HIA practically using the NHIS in Ghana as a case study.

## Carrying Out a Community HIA Practically: A Case Study of NHIS in Ghana

The ideas presented in this section were part of a PhD project where HIA was used to study the NHIS in Ghana practically.^27^ The findings are reported elsewhere, but the methodological concepts of carrying out a community HIA are recounted. Health impact assessment is a combination of procedures, methods, and tools by which a policy, program, or project may be judged as to its potential effects on the health of a population, and the distribution of those effects within the population.^11,28^ The practical application of HIA to the NHIS followed the conventional desk–based HIA processes with modification and additions.

**Figure 1.**
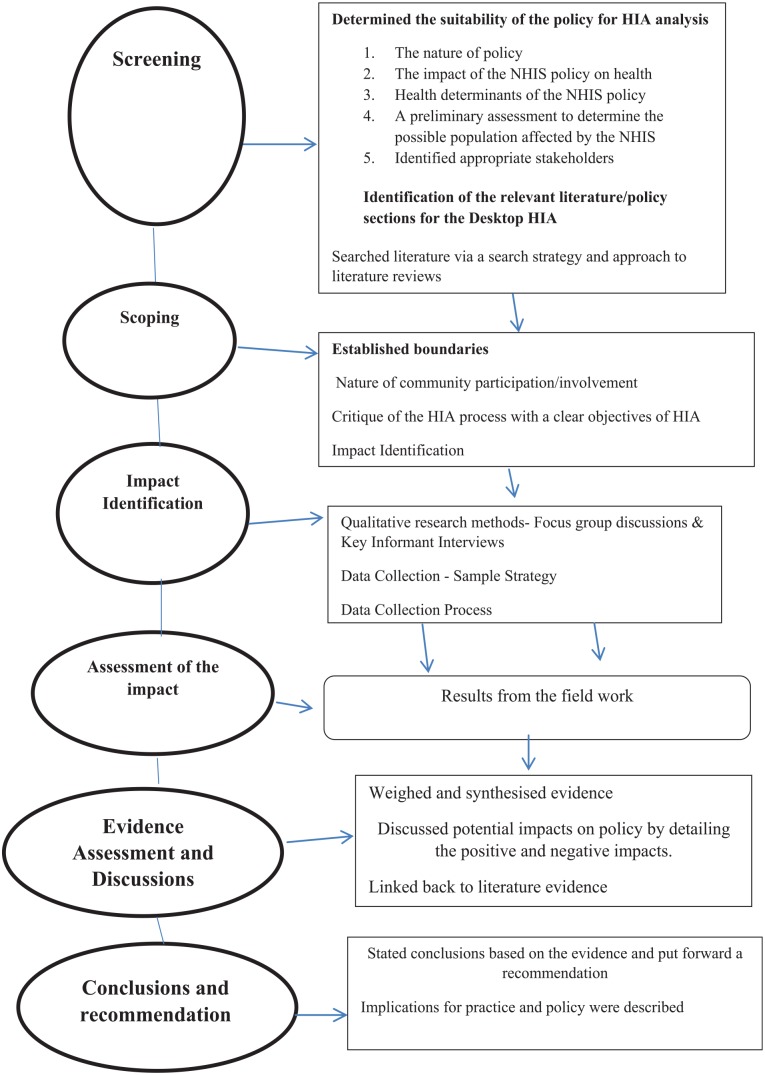
Steps and processes. *Note.* HIA = health impact assessments; NHIS = National Health Insurance Scheme.

Initially, there was a need to search whether the NHIS policy or program in Ghana had an influence on health and what kind of influence. At the screening phase of the HIA process, it was learnt that additional evidence was needed, so a scoping process was carried out to ascertain what auxiliary work ought to be carried out, by whom, and how. Second, methods established were followed by impact identifications of the HIA process by reporting on community processes including results, then appraisal of appropriateness of the report, and finally by action to modify the proposed policy, program, or project. The outcomes of any of these phases needed reassessment of former phases (see Figure 1).

## Method

A comprehensive review of the NHIS literature was identified at the screening phase by the desk-based HIA. This was followed by a community field work as part of qualitative research. The qualitative study of the NHIS in Ghana brought to bear the community perspectives of HIA. The results of the desk-based HIA are reported elsewhere, but the processes are found in Figure 1. The qualitative aspects of the HIA process are referenced in this article to show how community HIA may be practically conducted, especially in the African context. The qualitative aspect of the HIA explored the views of respondents via focus groups and key informants interview to gather data from communities in Ghana to support evidence in literature. The literature review process at the HIA screening phase is first described, followed by the processes of key informant interviews and FGDs.

## Literature Review

An extensive and systematic search of the literature was conducted on various academic databases to locate literature on HIA and NHIS at the screening phase (Figure 1). The search strategy was devised in collaboration with the NHIS database. During the literature review process, a search for evidence was made from published and unpublished research work on the NHIS policy, databases, newspapers, and other forms that were identified as significant features of the policy problem. Apart from the manual search, the search processes were streamlined with key words and possible synonyms in each of the search terms used. The bibliographical databases (including Medline, applied social science index, academic search complete, EBSCO, PubMed, etc) were searched electronically. Additional materials were obtained through a hand search of the reference lists of articles located by the electronic searches. Moreover, unpublished materials, including studies in progress and those undertaken at regional and health authority level without intention to publish, were sought. All citations were in English and, to ensure that the articles were up to date, citations were limited to articles published between 2000 and 2016. The results of this literature search are reported elsewhere. This article details the processes of carrying out community HIA. The next phase after the screening was scoping where community perspectives were explored by focus groups and key informant’s interviews. The focus groups and key informants enhanced involvement of the community. Community involvement parameters were set at the scoping phase (Figure 1). Scoping sets boundaries and establishes a pathfinder for undertaking an HIA.^29,30^ Community involvement is discussed below.

## Community Involvement

After the screening phase suggested potential impacts of the NHIS policy on the Ghanaian population, it became essential to engage the community in FGDs and Key Informant interviews to listen to personal experiences and opinions of NHIS. These discussions then supported data obtained from the literature. The FGDs and key informant interviews were done as part of the qualitative research process. Community involvement is essential in the development of policies that impact on the health of a society.^31^ Thus, engaging the community in the qualitative research process was essential and therefore the opinions, experiences, or expectations of the communities were considered through consultations in FGDs and interviews with key informants.

## Why Focus Groups and Interviews?

The primary aim of the study was to analyze the NHIS using HIA and the purpose of the focus groups and interviews was to facilitate community involvement as part of the qualitative study. First, grassroots Ghanaians were engaged in an FGD (considered in the data collection section). The FGDs allowed engagement with the community and listened to people’s experiences and opinions of NHIS policy to support data from the literature. The FGDs were chosen for this study because of the brainstorming format that continues to be one of the best means of gaining in-depth opinions and feedback on a broad range of issues.^32^

Second, community leaders, the elite and key informants on the NHIS policy, were engaged through face-to-face interviews. From an epistemological viewpoint, Moss and Shank^33^ note that qualitative interviews are appropriate for seeking access to respondents’ understanding of their social world. Interviews are one of the generally accepted, identified, and indispensable mechanisms for collecting evidence or data for social science research.^34^ As the topic on NHIS is about studying human affairs, the interviews provide a useful source of information and they stand the chance of obtaining rich information from their informants.^35^

In total, the FGDs and the interviews were for the grassroots Ghanaians and key informants to explore their experiences, thoughts, and feelings because they are at the receiving end of NHIS services with direct and indirect impact. The data collection processes including sampling of the FGDs and interviews respondents are detailed below as part of the impact identification process in HIA (Figure 1).

## Impacts Identification

As indicated in Figure 1, this process involves collecting information (data and evidence) to identify the potential and/or actual impacts of the NHIS policy on people. The core activities in this stage of the study were gathering evidence from the Ghanaian communities, engaging with key stakeholders and target groups to gather data, gathering evidence on the impacts of the NHIS intervention through qualitative methods within HIA (see Figure 1). Mahoney et al^20^ note that qualitative methods within HIA dwell on community involvement and comprise a range of activities such as interviews and focus groups. Qualitative methods have been reported to be distinctive to individual experiences, perceptions, beliefs, and understandings and also answer the primary research questions, thus justified. The qualitative data collection processes then followed are narrated below.

## Data Collection—Sample Strategy

The community selection processes are discussed first, followed by selection of household processes. The sample strategy aimed to select communities that would bring out variations in the findings. We chose the areas that could offer interesting findings such as those towns and villages that had varied economy and geography. We deliberately selected 7 differing communities from the Ghanaian communities in the Ashanti region areas. The 7 communities selected were justified because they were based on their distinctive geographic characteristics, portraying characteristics of the rural areas. Two communities were well developed and affluent. Another 2 communities were both slightly undeveloped and developed and were popularly known as middle-class areas. The remaining 3 communities, on the other hand, were overpopulated and underdeveloped communities. They are deprived and marginalized communities with serious socioeconomic problems—in short, these were very poor communities. Local authorities (chiefs, assemblymen, opinion leaders) of these communities were approached to introduce ourselves and explain the purpose of the study. The authorities of the hospital in the town were also informed about the project and the recruitment of participants. When permission was given to recruit from households for the focus groups, invitation letters were distributed to various households from urban to rural communities as described below. For each selected community, on approaching the first house, a coin was tossed. A head meant this house was taken as the first in the counting process and then the fifth house was selected for inclusion. A tail meant that the second house was considered as number one in the counting process. The fifth house from this was then selected for inclusion. This was systematic random sampling. Therefore, every fifth house was selected with the view of inclusion and this continued until saturation. Information about the nature, purpose, and objectives of the study were provided to the invitees in the selected households and their verbal/signature consent was obtained during the FGD.

## Data Collection Process

Data were collected between July and October 2015 from residents of the Ghanaian communities in the Ashanti Region. Participants in the focus groups were aged over 18 years and were from the sample households within the communities of the district. The sampling unit of the focus groups was the household, which is defined as “a person or group of persons living together in the same house or compound, sharing the same housekeeping arrangements.”^36^

## Focus Groups Data Collection

The sampling unit of the FGD was household and most of the participants in each group discussion were quite familiar with each other and were living together in a large compound house, in a small family house, or in the community. Krueger^37^ averred that having such a close-knit group is useful, because people who are already acquainted through living, working, or socializing might normally discuss (or evade) the sorts of issues likely to be raised in the FGD sessions. Thus, a naturally occurring group is one of the most important settings in which ideas are formed and decisions made. Study participants were free to terminate their participation at any time, even after the focus group had started. Participants in the focus groups were engaged in the discussions via an open-ended semi-structured questionnaire in a local language. The questions were reviewed occasionally to marry the aim and objectives of the project. The FGDs were conducted in parallel with the face-to-face interviews of key informants during the data collection period. The interview methods are discussed below.

## Key Informant and Elites Interviews Data Collection

**Figure 2.**
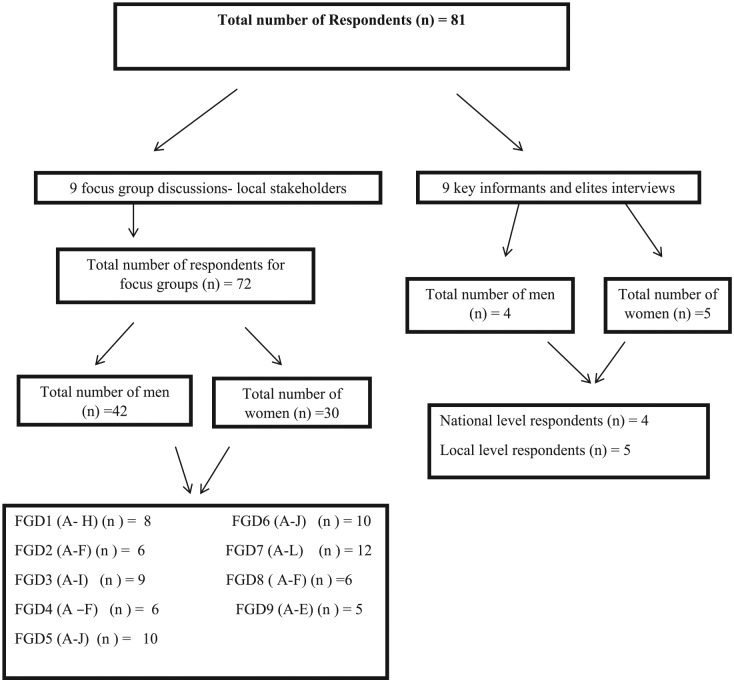
Sample overview/characteristics. *Note.* FGD = focus group discussion.

Nine key informant interviews (Figure 2) enabled the gathering of information from policymakers, officials from the NHIS and government, Ghana Health Service, Ministry of Health, and service providers on the impact of the NHIS policy on the wider community. According to Warheit et al,^38^ key informants are used to gather detailed and rich evidence in a relatively easy and inexpensive way. Denzin and Lincoln^39^ suggest that such interviews look like a normal everyday conversation, allowing for a free flow of ideas and information. Morris^40^ explains that key informant interviews also allow the investigator to establish relationships with the respondents and to clarify questions, thus providing an opportunity to build or strengthen relationships with important community informants and stakeholders. Overall, all the 9 face-to-face key and elite informant interviews provided a free exchange of ideas, with more complex questions asked and detailed responses of impact of the NHIS received.

## Semi-Structured Interview Schedules

The specific research question that guided the qualitative component of the study was: *what is the impact of the NHIS policy on the people of Ghana?* Semi-structured interviews were determined to be suitable methods because they allow discussions of the concepts in depth and they accommodate changes in the interview protocol in line with the emergence of new themes and the specific areas of expertise of participants. A semi-structured interview guide that varied slightly depending on the category of stakeholder was used. In general, it included questions on the impact of the NHIS on the population of Ghana and health care finance and the informants’ role in development, implementation, and decision-making pertaining to policy. Forty-five-minute to 1-hour face-to-face interviews were conducted with all the agreed participants. Interviews were held in either homes or offices and no one was coerced to participate. All participants were promised confidentiality and anonymity, and everyone provided oral/written consent.

## Ethics and Tape Recording

The study was approved by the ethics committees of our Ghanaian and United Kingdom’s institutions respectively.

A tape recorder was used to document both the key informant interviews and the FGDs. The tape recording approach allowed us to engage freely in the conversation without worrying about note-taking. The recorded information helped when we were transcribing the data. Brief notes were also taken during the interview, written down, and organized at the end of the interview. These notes were used to fill in information gaps as well as to supplement the recorded conversation. Consents were obtained from the key informant and the focus group participants to audiotape the interview/FGDs. The audio taping was discussed with all the participants before scheduling the interview appointments.

## Results

Some 72 individuals participated in 9 FGDs including 42 men and 30 women. Nine key informants were also engaged in an interview (Figure 2 shows how the samples were framed). The data were analyzed together using thematic network approach. Appraisal of the results as part of HIA processes were carried out at original PhD work,^27^ but it should be noted that this article is focused on the processes and the methods of carrying out Community HIA; hence, the appraised results is not discussed (see Figure 1). In this article, we describe below the concepts and methods of community HIA.

## Discussion

The community HIA concepts and methods have been narrated. First, the community involvement brought about networking and built links with the Ghanaian communities. Having initiated contacts, responded, and interacted with individuals and key informants, links were built between the leader of FGDs and the communities in a number of ways. Second, the leader of FGDs acted as access points for the poor individuals in the communities. Third, the participants in the FGDs and the key informants had a role in support of the participatory process. An important part of the community involvement was about supporting the involvement of poor individuals who were traditionally under-represented. This study has proposed how a likely concept and method of community HIA in the African context can be conducted. It incorporates community perspectives of FGDs and interviews in HIA methods.

By considering the range of factors involved in community HIA, the developed HIA concept and method incorporate the broad parameters of grassroots and key informants which influence HIA approaches and practice. In the public policy arena, the broad context in which HIAs are undertaken have been shown to be desk based. This community perspective incorporated in the HIA has shown that HIA can include local and key actors (the elite stakeholders and lay people involved in policy-making). This is in line with the 5 fundamental principles of HIA described in HIA literature—sustainability, human rights, equity, ethical application of evidence, and collaboration (see Table 1).

Community HIA is concerned with the health impacts of future, as well as present, and takes into account lay views to support the health, development, and well-being of populations in decision-making (see Table 1). People are at the center of concerns for sustainable development and their views cannot be ignored in issues that affect them. Decision-making power should be decentralized, with accountable decisions being made as close to an individual citizen as possible. In the context of community HIA in Africa, this means decisions about the approval of planned interventions, or conditions under which they might operate, should be taken as close to the affected people and communities as possible, with local people having an input into the approval and management processes to emphasis short- and long-term goals.

Community HIA is concerned with human rights as discussed above and most nations have endorsed at least one global accord that comprises the right to health (see Table 1). The Universal Agreement on Financial, Social and Cultural Rights comprises one of the extensive related provisions on human rights.^23^ Countries that are part of this agreement appreciate the rights of citizens to enjoy the highest attainable standard of mental and physical health. In any event, the special status of the UDHR as the premier human rights instrument in content and authority allows us to assert that its core principles have crystallized into *jus cogens principles* or peremptory norms of customary international binding on all states.^41^

Hunt and MacNaughton^42^ argue that the health determinants that underpin the right to the highest attainable standard of mental and physical health are food, community participation, transportation, and equity. According to Scott-Samuel and O’Keefe,^10^ it is a right to influence choices that affect a person’s health. In recent years, Marmot et al^6^ and CSDH^22^ have expanded human rights with progressive understanding to mean that countries must take obvious steps toward recognizing the right of health for all and retrogressive steps are acknowledged as not acceptable in health. Thus, community HIA are good tools for guaranteeing progressive understanding in policy improvements.

Community HIA is also underpinned by an explicit value system and a focus on social justice in which equity plays a major role so that not only both health inequalities and inequities in health are explored and addressed wherever possible.^42^ The World Health Assembly according to Marmot et al^43^ accepted a declaration to address health inequalities that was influenced by the CSDH report.^22^ This, however, gave the WHO a better obligation to promote HIA.

Moreover, community HIA can foster collaboration as indicated in the concepts and methods discussion above. Joining synergies across sectors and the importance of investing in health beyond the narrow health services sector is crucial in community HIA. The opinions and views of experts should not be the sole consideration in decisions about planned interventions. This is probably the main area where Community HIA can help to maximize multisectoral approaches by incorporating lay views. Involving communities in practices in methods within HIA help make policy initiatives more practical because it makes community HIA processes engage with both the people who are affected by the policy and the key stakeholders within the community.^44^ Focus groups and key interviews fit most closely within community HIA, with the conception of community HIA to be consultative.^11^ It is most appropriate to undertake this approach when community involvement is only one aspect of a wider information strategy within the HIA, where each component is valued equally. This type of HIA seeks only to consult and does not raise community expectations as to the outcome of their involvement but can raise community awareness around certain issues and increase confidence in understanding the policy. Lay community views have been reported to be distinctive to individual experiences, perceptions, beliefs, and understandings.^11^ Focus groups and key informants’ views within community HIA enhance participation and use similar methods to the consultative HIA, while seeking to distribute some degree of power to members of the community or a specific group, usually through workshops, steering committees, and advisory groups.

## Conclusions

The main purpose of this article was to show how community HIA might be conducted in the African context using a case study of NHIS project in Ghana. HIA has not progressed in Africa and specifically in Ghana at the same level as in other countries, eg, in Europe, America, or Australia. In future, detailed quantitative work can confirm and refine our proposed method and concepts of incorporating lay perspectives in HIA both in Ghana and in other contexts and countries response to develop an international HIA consensus that moves the field forward. In summary, focus groups and interviews via involving community respondents and key informants within HIA are clearly linked to the principles underpinning health promotion and the broader community development field.^45^ Recognizing lay views with community HIAs demonstrate the significance of activities for health and well-being of communities as well as providing flexibility by representing, responding, and adjusting to decision-making. Last, but by no means the least, community HIA provides for inclusiveness by involving and including lay views of people from certain groups of society or provides a platform for communities to be part of decisions that shape their lives and influence their health and well-being. Community HIA looks at unintended consequences, spin-offs, or side-effects and can practically incorporate lay and expert views as demonstrated throughout the article. Community HIA can be conducted in other settings aside from Africa to continuously measure its success, effect or influence and estimation of size, quality, and value.
